# *Prunus persica* Crop Management Differentially Promotes Arbuscular Mycorrhizal Fungi Diversity in a Tropical Agro-Ecosystem

**DOI:** 10.1371/journal.pone.0088454

**Published:** 2014-02-10

**Authors:** Maria del Mar Alguacil, Emma Torrecillas, Zenaida Lozano, Maria Pilar Torres, Antonio Roldán

**Affiliations:** 1 CSIC-Centro de Edafología y Biología Aplicada del Segura, Department of Soil and Water Conservation, Campus de Espinardo, Murcia, Spain; 2 Universidad Central de Venezuela (UCV), Facultad de Agronomía, Instituto de Edafología, El Limón, Campus Universitario, Maracay, Venezuela; 3 Departamento de Biología Aplicada, Area de Botánica, Universidad Miguel Hernández, Elche, Alicante, Spain; The Pennsylvania State University, United States of America

## Abstract

Due to the important role of arbuscular mycorrhizal fungi (AMF) in ecosystem functioning, determination of the effect of management practices on the AMF diversity in agricultural soils is essential for the sustainability of these agro-ecosystems. The objective of this study was to compare the AMF diversity in *Prunus persica* roots under two types of fertilisation (inorganic, with or without manure) combined with integrated or chemical pest management in a Venezuelan agro-ecosystem. The AM fungal small-subunit (SSU) rRNA genes were subjected to PCR, cloning, sequencing and phylogenetic analyses. Twenty-one different phylotypes were identified: 15 belonged to the genus *Glomus,* one to *Claroideoglomus*, two to *Paraglomus*, one to *Acaulospora,* one to *Scutellospora* and one to *Archaeospora.* The distribution of the AMF community composition differed as a consequence of the treatment effects. The treatment combining organic and inorganic fertilisation with chemical pest control had the highest AMF richness and the treatment combining inorganic fertilisation with chemical pest had the lowest. The real causes and effects of these differences in the AMF community are very difficult to establish, since the crop management regimes tested were composed of several interacting factors. In conclusion, the crop management practices can exert a significant influence on the populations of AMF. The treatment combining organic and inorganic fertilisation with chemical pest control appears to be the most suitable agricultural management strategy with respect to improving the AMF diversity in this crop under tropical conditions, and thus for maintaining the agricultural and environmental sustainability of this agro-ecosystem.

## Introduction

The soil is a complex matrix containing microorganisms that play a key role in the functioning of terrestrial ecosystems. They mediate many processes, including nutrient cycles, organic matter decomposition, soil aggregate formation and plant performance. Arbuscular mycorrhizal fungi (AMF) are among the most-important soil microorganisms, being obligate symbionts in the roots of most land plants in both natural and agricultural ecosystems, where they increase plant uptake of mineral nutrients, especially phosphorus [1]. Other beneficial effects of AMF are plant growth promotion [2], increased tolerance of drought [3], heavy metals [4] and plant protection agents [5]. In fact, in a previous study carried out at the site that is also the subject of the current work [6], it was found that galls produced in *Prunus persica* roots due to infection with *Meloidogyne incognita* were extensively colonized by AMF, whose function might be to act as protection agents against opportunistic pathogens. Furthermore, the diversity of AMF influences a number of important ecosystem processes, including plant productivity, plant diversity and soil structure [7], [8], [9].

Due to the important role of AMF in ecosystem functioning, knowledge of the diversity of the AMF colonising the roots of crop plants in agricultural soils is essential for sustainable management of these agro-ecosystems. Fertilisation is a common practice used to increase the nutrient availability to crops and hence their yields. Studies carried out in recent years have considered the effect of different fertilisation treatments and cropping systems on AMF diversity. Thus, a general decrease in AMF diversity has been found with the use of mineral fertilisers [10], [11], [12], [13], [14], although not in all cases [15]. Others studies showed that fertilisation with manures stimulated the AMF populations [13], [16], [17], but studies on the species composition of the AMF community colonising crop roots in response to other management practices are scarce [18], [19], [20], [21].

*Prunus persica* (L.) Batsch. (peach) is a fruit tree, native to Asia, introduced into Venezuela. Peach production in Venezuela is an activity that generates steady employment and is aimed primarily at the domestic market [22]. At present, there are 2,500 hectares that can produce more than 15,000 metric tons of fruit. Peach production in Venezuela is a cropping system in which fertilisers and pest control are combined in order to maximise yields while maintaining a suitable soil nutrient content.

Due to the economic importance of this fruit crop, the elucidation of whether there is a fertiliser/pest management combination that can maintain or increase the AMF diversity colonising the roots is an important step towards sustainable soil use and therefore protection of biodiversity. Therefore, the objective of this study was to compare the AMF diversity in *P. persica* roots under two fertilisation treatments (inorganic, with or without manure) combined with integrated or chemical pest management.

## Materials and Methods

### Ethics Statement

No specific permits were required for the described field studies since these locations are not privately-owned or protected in any way. Field studies did not involve endangered or protected species.

### Study site and Sampling

The study was conducted in a *P. persica* orchard located at the “Colonia Tovar” Aragua State, in the north of Venezuela (latitude 10° 29' N, longitude 67° 07' W, 1790 msl). The climate is tropical temperate (mean annual temperature of 16.8 °C, annual average rainfall of 1271 mm). The soil was classified as a sandy loam Inceptisol [23]. The soil characteristics were: pH of 5.18, 5.75% clay, 40.5% silt, 53.75% sand, 6.46 cmol kg^−1^ of cationic exchange capacity, Total N 2.7 g kg^−1^, Available P 32 µg g^−1^, 5.9% organic matter and bulk density 1.29 g cm^−3^

The plants used in this survey were 13-year old peach (*Prunus persica* (L). Batsch cv. Criollo Amarillo). The experimental sampling was a randomised block design with two factors and four replication blocks (100 m^2^ each) in an experimental area of approximately 1000 m^2^. The first factor consisted of two types of fertilization and the second factor was two different procedures of pest control. Four treatments were established in the sampling design. The treatments were selected in order to provide more sustainable practices to producers, since the regular management practices are limited to exclusively use of inorganic fertilization and an excessive use of pesticides

T1: Combination of organic and inorganic fertilization (ComFert) and integrated pest management (IntM).

T2: Inorganic fertilization (InorgFert) and integrated pest management (IntM).

T3: Inorganic fertilization (InorgFert) and chemical pest control (ChemM).

T4: Combination of organic and inorganic fertilization (ComFert) and chemical pest control (ChemM).

-ComFert consisted of application of chicken manure (1400 kg ha^−1^), urea (140 kg ha^−1^), complex fertilizer (NPK) 12-12-17 (280 kg ha^−1^), and potassium sulfate (40 kg ha^−1^).

-InorgFert consisted of application of urea (140 kg ha^−1^), complex fertilizer (NPK) 12-12-17 (400 kg ha^−1^) and potassium sulfate (70 kgha^−1^).

-IntM consisted of weekly applications of *Beauveria bassiana* (300 g spores ha^−1^) for one month, subsequently weekly applications for two months of *Trichoderma harzianum* (300 g spores ha^−1^) and lastly applications every 15 days for two more months of *Trichoderma harzianum* (300 g spores ha^−1^). We applied these products as biocontrol agents against fungal diseases.

-ChemM consisted of applications of different chemicals from the beginning of flowering aimed at insect pests control and subsequently the incidence of foliar diseases. Thus, weekly applications for six weeks of Profenofos 0.6 kg a.i. ha^−1^ (Curacron ®) + Mancozeb 8 kg i.a. ha^−1^ (Dithane ®) were made. For control of *Oidium leucoconium,* was applied twice the mixture Urea 10 kg ha^−1^ + Flusilazol 0.4 kg a.i. ha^−1^ (Punch ®) + Mancozeb 4 kg a.i. ha^−1^ (Dithane ®), then weekly applications of Mancozeb 4 kg a.i. ha^−1^ (Dithane ®) + Profenofos 0.6 kg a.i. ha^−1^ (Curacron ®) + Endosulfuran 2.8 kg a.i. ha^−1^ (Thionil). For control of *Monilia cinerea* were performed four weekly applications of Carbendazin 2 kg a.i. ha^−1^ (Bavistin ®).

The treatments were applied for one year and sampling was conducted after fruit harvest (February 2011). Four plants (one per block) of each treatment were sampled providing 16 samples in total. The roots were sampled using three soil cores from three points/single tree/block.

### Root DNA extraction and PCR

All PCR experiments were run using DNA preparations consisting of pooled roots of individual plants. DNA extractions from 16 root samples were carried out.

For each sample (total 16), total DNA was extracted from (0.1 g) fine root material using a DNeasy plant mini Kit following the manufacturer’s recommendations (Qiagen). The roots samples were placed into a 2-ml screw-cap propylene tube together with two tungsten carbide balls (3 mm) and beaten (3 min, 13000 r.p.m.) using a mixer mill (MM 400, Retsch, Haan, Germany). The extracted DNA was resuspended in 20 µl of water. Several dilutions of extracted DNA (1/10, 1/50, 1/100) were prepared and 2 µl were used as template. Partial small-subunit (SSU) ribosomal RNA gene fragments were amplified using nested PCR with the universal eukaryotic primers NS1 and NS4 [24]. PCR was carried out in a final volume of 25 ml using the “ready to go” PCR beads (Amersham Pharmacia Biotech, Piscataway, N.J.), 0.2 mM dNTPs and 0.5 mM of each primer (PCR conditions: 94 °C for 3 min, then 30 cycles at 94 °C for 30 s, 40 °C for 1 min, 72 °C for 1 min, followed by a final extension period at 72 °C for 10 min). Two µl of several dilutions (1/10, 1/20, 1/50 and 1/100) from the first PCR were used as template DNA in a second PCR reaction performed using the specific primers AML1 and AML2 [44]. PCR reactions were carried out in a final volume of 25 ml using the “ready to go” PCR beads (Amersham Pharmacia Biotech, Piscataway, N.J.), 0.2 mM dNTPs and 0.5 mM of each primer (PCR conditions: 94 °C for 3 min, then 30 cycles of 1 min denaturation at 94 °C, 1 min primer annealing at 50 °C and 1 min extension at 72 °C, followed by a final extension period of 10 min at 72 °C). Positive and negative controls using PCR positive products and sterile water respectively were also included in all amplifications. All the PCR reactions were run on a Perkin Elmer Cetus DNA Thermal Cycler. Reactions yields were estimated by using a 1.2% agarose gel containing GelRed™ (Biotium).

### Cloning and sequencing

The PCR products were purified using a Gel extraction Kit (Qiagen) cloned into pGEM-T Easy (Promega) and transformed into *Escherichia coli* (XL2-Blue). Thirty two positive transformants were screened in each resulting SSU rRNA gene library, using 0.8 units of RedTaq DNA polymerase (Sigma) and a re-amplification with AML1 and AML2 primers with the same conditions described above. Product quality and size were checked in agarose gels as described above. All clones having inserts of the correct size in each library were sequenced.

Clones were grown in liquid culture and the plasmid extracted using the QIAprep Spin Miniprep Kit (Qiagen). The sequencing was done by Laboratory of Sistemas Genómicos (Valencia, Spain) using the universal primers SP6 and T7. Sequence editing was done using the program Sequencher version 4.1.4 (Gene Codes Corporation). Unique sequences of the clones generated in this study have been deposited at the National Centre for Biotechnology Information (NCBI) GenBank (http://www.ncbi.nlm.nih.gov) under the accession numbers HE613450 to HE613504.

### Phylogenetical analysis

Sequence similarities were determined using the Basic Local Alignment Search Tool (BLASTn) sequence similarity search tool [25] provided by GenBank.

Phylogenetic analysis was carried out on the sequences obtained in this study and those corresponding to the closest matches from GenBank as well as sequences from cultured AMF taxa including representatives of the major taxonomical groups described by Schüßler et al. [26]. Sequences were aligned using the program ClustalX [27] and the alignment was adjusted manually in GeneDoc [28]. Neighbour-joining (NJ) [29] and maximum likelihood (ML) phylogenetic analyses were performed with the programs PAUP4.08b [30] and RAxML v.7.0.4 [31], respectively. The evolutionary distances for the NJ tree were computed using the maximum composite likelihood method with 1000 bootstrap replicates. For the ML analysis, a GTR-GAMMA model of evolution was used. The ML bootstrap values were calculated with 1000 replicates using the same substitution model. *Endogone pisiformis* Link and *Mortierella polycephala* Coem, were used as the out-groups.

### Statistical analysis

Treatments effects on the number of phylotypes per root sample were compared using analysis of variance and comparisons among means were made using the Duncan’s test calculated at P<0.05. The effect of two factors: types of fertilizer and pest management on AMF community composition were tested using a two-way analysis of variance, The statistical procedures were carried out with the software package SPSS 19.0 for Windows.

Canonical-correspondence analysis (CCA) with the relative abundance of clones per AMF sequence types found in *P. persica* roots under different treatments was performed. The results were summarized in an ordination diagram conducted in CANOCO for Windows v. 4.5 [32]. CCA is a multivariate statistical method that allows comparisons of AM fungal community compositions between four treatments.

The Shannon-Weaver (H’) index was calculated as an additional measure of diversity, as it combines two components of diversity, i.e., species richness and evenness. It is calculated from the equation H’ =  − ∑*p_i_*(ln *p_i_*), where *p_i_* is the proportion of individuals found in the *i*th species (in a sample, the true value of *p_i_* is unknown but is estimated as *n_i_*/*N*, [here and throughout, *n_i_* is the number of individuals in the *i*th species; N is the total number of individuals of all species]).

The number of clones for each AMF phylotypes in each soil sample was used to construct the sampling effort curves (with 95% confidence intervals) using the software EstimateS 8.00 [33]. The sample order was randomized by 100 replications.

## Results

### Sequence identity and phylogenetic analysis

In our study, 16 root samples (four repetitions per treatment) that were subjected to DNA extraction produced clonable PCR products of the expected size (about 795 bp). Overall, 512 clones from 16 clone libraries were screened by PCR; out of these, 449 contained the small-subunit rRNA gene fragment and an average of 15 clones per root sample were sequenced (in total, 242 sequences). According to the BLAST search in GenBank, sequences with a high degree of similarity (98−99%) to taxa belonging to the phylum Glomeromycota were produced by 227 clones. The remaining 15 clones produced incomplete sequences.

**Figure 1:**
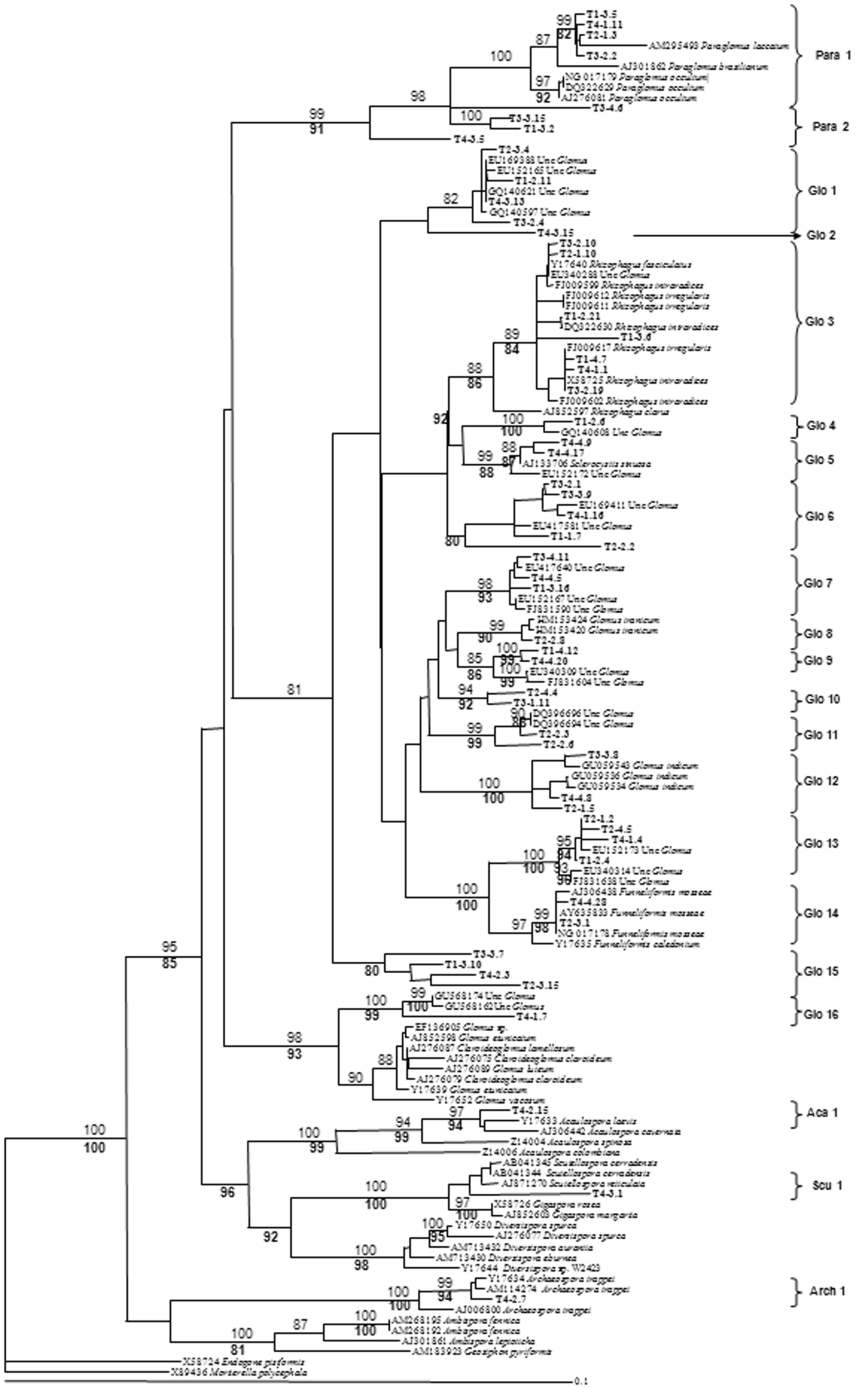
Phylogenetic tree of AMF sequences isolated from the *Prunus persica* roots under different treatments (T1: Combination of organic and inorganic fertilization and integrated pest management; T2: Inorganic fertilization and integrated pest management; T3: Inorganic fertilization and chemical pest control; T4: Combination of organic and inorganic fertilization and chemical pest control), reference sequences corresponding to the closest matches from GeneBank as well as sequences from cultured AMF taxa including representatives of the major taxonomical groups. Numbers above branches indicate the bootstrap values determined for Neighbour-Joining (NJ) analysis; bold numbers below branches indicate the bootstrap values of the maximum likelihood analysis. Sequences are labelled with the number of treatment from which they were obtained (T1, T2, T3, T4) and the clone identity number, Group identifiers (for example Glo 1) are AM fungal sequences types found in our study. Since identical sequences were detected, the clones producing the same sequence for each treatment were represented once in the alignment for clarity (Table S1 in File S1 material show a detailed description of the total number of clones of each AMF phylotype that were recovered from each treatment).

The 227 sequences were grouped in 21 different AMF sequence types or phylotypes, with sequence similarities varying from 97 to 100% and bootstrap values ≥ 80% (Fig. 1). These phylotypes were grouped in five families: the Glomeraceae, Paraglomeraceae, Acaulosporaceae, Gigasporaceae and Archaeosporaceae. Sixteen of these sequence groups belonged to the genus *Glomus,* two to the genus *Paraglomus*, one to the genus *Acaulospora,* one to the genus *Scutellospora* and one to the genus *Archaeospora*.

**Figure 2:**
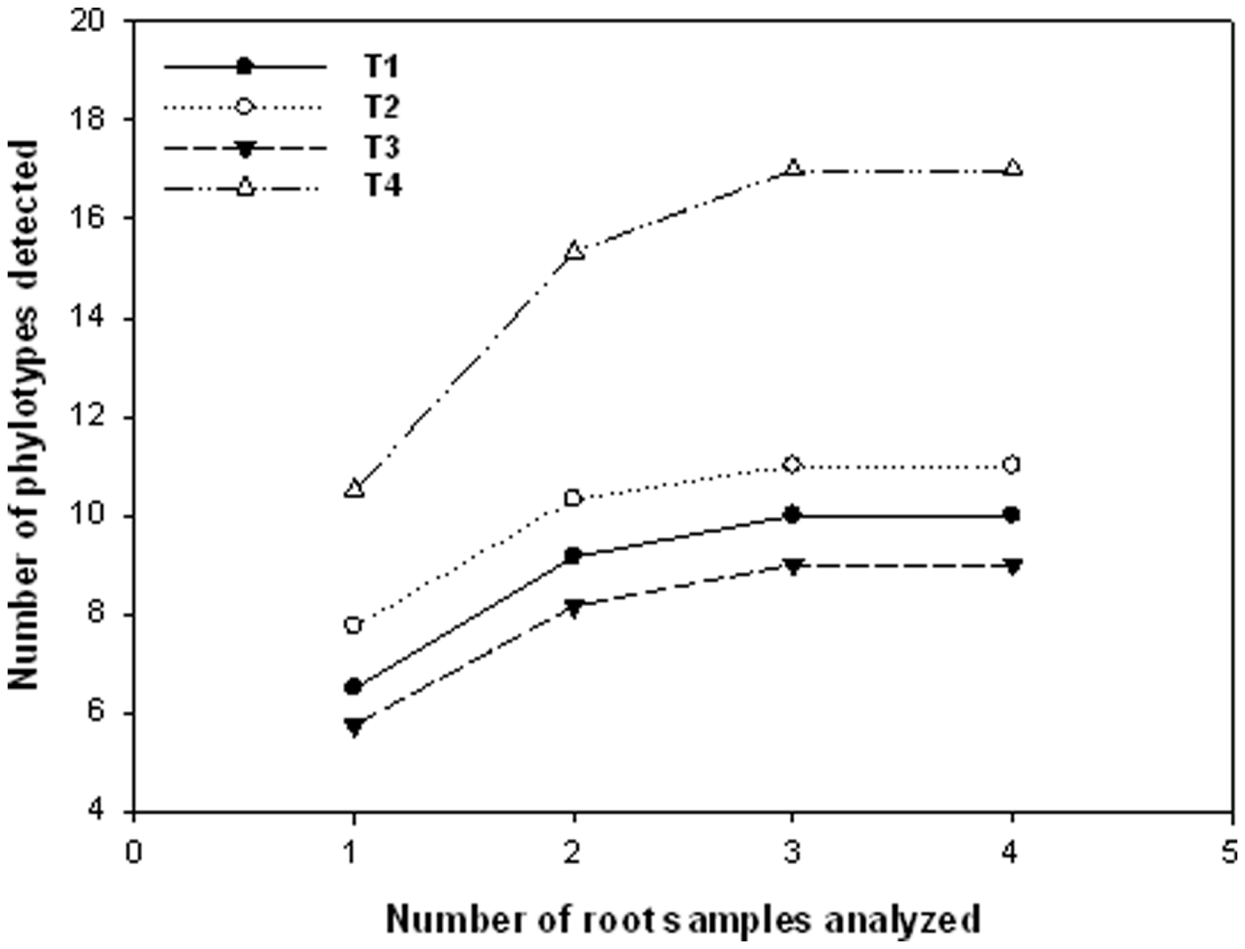
Sampling effort curves for *Prunus persica* roots under different treatments analysed. T1: Combination of organic and inorganic fertilization and integrated pest management; T2: Inorganic fertilization and integrated pest management; T3: Inorganic fertilization and chemical pest control; T4: Combination of organic and inorganic fertilization and chemical pest control. The number of clones for each AMF phylotypes in each root sample was used to construct the sampling effort curves (with 95% confidence intervals) using the software EstimateS 8.00 (Colwell, [33]). The sample order was randomized by 100 replications.

For the number of clones sequenced, the sampling effort curves showed a decreasing rate of accumulation of phylotypes, reaching the asymptote (Fig. 2). This pattern indicates that the clones analysed covered the AMF diversity colonising the *P. persica* roots under the four treatments. Therefore, no more clones were sequenced.

Nine phylotypes corresponded to morphologically-defined species: six were related to sequences from single, morphologically-described species (Para 1 to *Paraglomus laccatum,* Glo 5 to *Sclerocystis sinuosa,* Glo 8 to *Glomus iranicum,* Glo G12 to *Glomus indicum*, Glo 14 to *Funneliformis mosseae* and Arch 1 to *Archaeospora trappei*) and three were related to sequences belonging to two or three different, morphologically-described species (Glo 3 was related to a species group including *Rhizophagus intraradices/irregularis/fasciculatus*, Aca 1 to an *Acaulospora cavernata/laevis* group and Scu 1 to a *Scutellospora cerradensis/reticulata* group). Eight phylotypes were related to uncultured glomalean species that have not been characterised morphologically (Glo 1, Glo 4, Glo 6, Glo 7, Glo 9, Glo 11, Glo 13 and Glo 16) and the remaining four phylotypes were not related to any sequences of AMF in the database (Glo 2, Glo 10, Glo 15 and Para 2) (Fig. 1).

The AMF community composition

There were significant differences in the AMF taxon richness. The ComFert+ChemM treatment harboured the highest mean number of AMF phylotypes per root sample (8.00), which was significantly different from the value for the InorgFert+ChemM treatment (4.25) according to Duncańs multiple-comparison test. The mean number of AMF phylotypes detected in the tree roots receiving the ComFert+IntM or InorgFert+IntM treatments was the same (5.75 ) and no significant differences between these and either of the treatments mentioned above were found.

The factorial analysis showed that both factors: fertilization and pest management had a significant effect on the AMF community composition (*p* = 0.002, *F* = 9.734 and *p* = 0.007, *F* = 7.412, respectively) The interaction between these factors was not significant (*p* = 0.906, *F* = 0.014).

**Figure 3:**
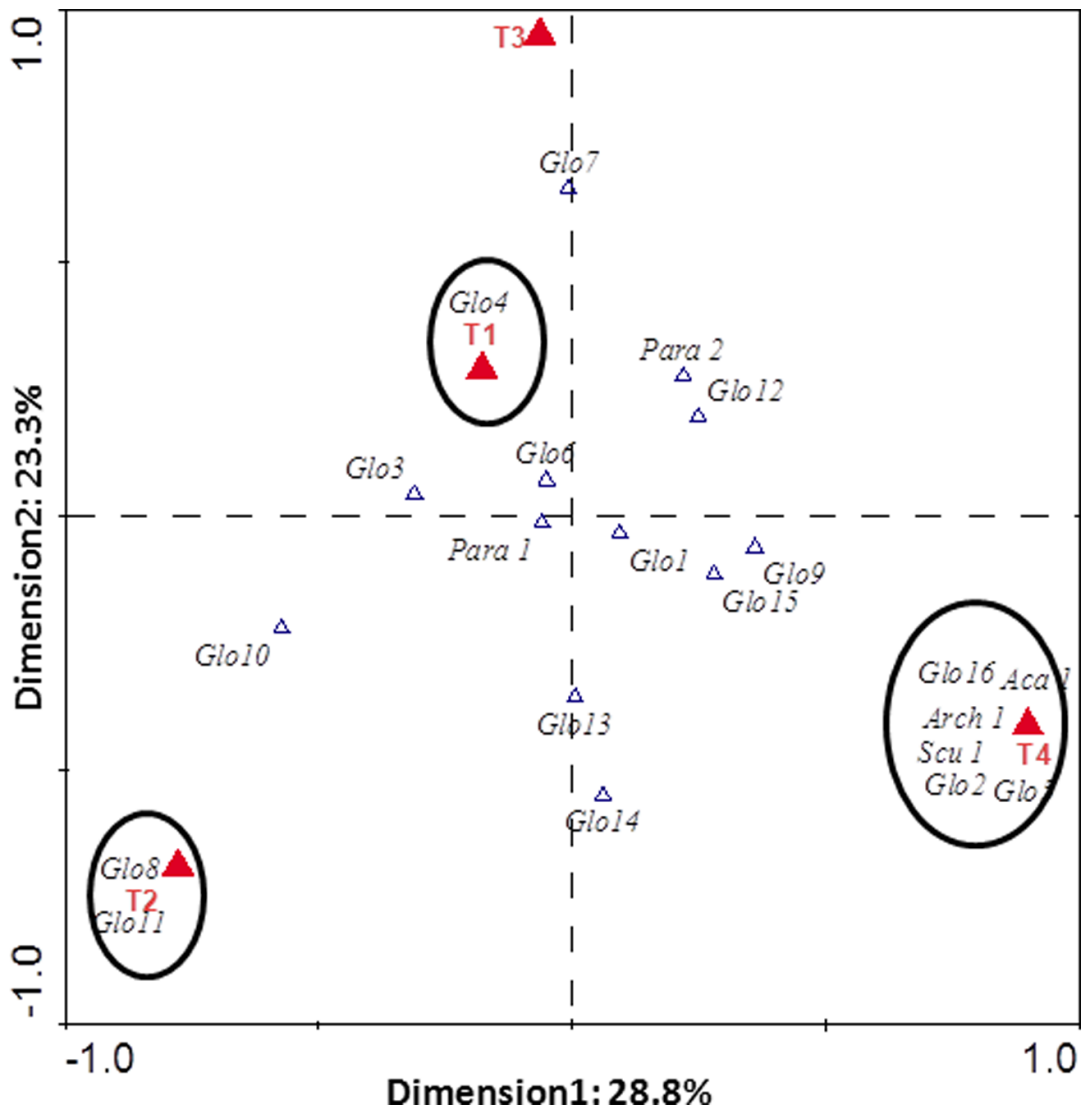
Canonical Correspondence analysis (CCA) of the AM fungal community composition found in the roots of *P. persica* under different treatments (T1: Combination of organic and inorganic fertilization and integrated pest management; T2: Inorganic fertilization and integrated pest management; T3: Inorganic fertilization and chemical pest control; T4: Combination of organic and inorganic fertilization and chemical pest control). Full triangles represent the treatments and the open triangles the AMF phylotypes. Open circles represent the AMF phylotypes exclusively found in individual treatments.

The AMF communities of tree roots in the InorgFert+ChemM treatment had the lowest diversity (*H* ´ = 1.78), with the lowest total number of AMF sequence types (9). The trees from the ComFert+IntM and InorgFert+IntM treatments had similar AMF diversity (H≈2.0), while the treatment ComFert+ChemM yielded the highest number of different AMF sequence types (17) and showed the highest diversity index (*H* ´ = 2.69). In the CCA diagram (Fig. 3), the different distributions of the AMF phylotypes, as a consequence of the treatments, can be observed. The symbols representing different treatments are distant to each other, which demonstrates that the treatments had a significant effect on the AMF community composition, with the different treatments hosting distinct phylotypes. This diagram also shows the AMF phylotypes found exclusively in each treatment.

## Discussion

In this study we compared the diversity of AMF in *Prunus persica* roots under two types of fertilisation (inorganic, with or without manure) combined with integrated or chemical pest management in a tropical agro-ecosystem.

It is worth noting the high number of phylotypes found in this study (twenty-one) in comparison with other studies carried out also in agricultural soils. Daniell et al. [34] and Helgason et al. [35] found 10 phylotypes in arable soils around North Yorkshire, U.K. Toljander et al. [36] found eight phylotypes, all belonging to the genus *Glomus,* in a field experiment using different organic and mineral fertilisers, while Hijri et al. [37] found 10 phylotypes in a conventional maize field in Germany. Also Verbruggen et al. [38] found that arbuscular mycorrhizal fungi richness varied from one to 11 phylotypes among organically and conventionally managed fields in the Netherlands. Alguacil et al. [39] found nine phylotypes in a study carried out in tropical savanna soils planted with leguminous forage under different doses of phosphorus fertiliser.

We observed different AMF community composition between treatments. Some studies have pointed out that soil physical-chemical properties, such as pH and nutrient content, are the factors influencing the structure of AMF communities in agricultural systems [17], [36], [40], [41], [42], [43], [44]. In our study, we did not find significant differences in soil characteristics among treatments (Table S2 in File S1), so the observed differences in the number of AMF phylotypes or AMF richness can be attributed to the different treatments applied. It has been reported that the AMF diversity is higher in soils amended with different organic substrates (or that the AMF diversity increases in organically-managed soils) [13], [17], [36], [45], [46]. Moreover, the addition of chemical pest-control products could have prevented the colonisation by non-AM fungi inside the roots, favouring the most-tolerant AMF species.

In contrast to some studies which indicated that species of the *Paraglomeraceae* appear to be rare or poor in agricultural soils [37], [47], [48], we found that Para 1 was one of the most-abundant groups in our study (12.8% of the AMF clones analysed), together with Glo 1 (15.9%) and Glo 3 (17.6%) (Table 1); Glo 6 and Glo 15 were found in all treatments, but their occurrence frequency was low (<6.2% of clones). The presence of the genus *Paraglomus* has been observed also in other agricultural management studies using group-specific primers [37], [49] or the same pair of primers as ourselves [50]. In the case of Glo 3 (related to the *R. intraradices/irregularis* species complex group), our results are in accordance with several studies carried out in agricultural soils where this AMF taxon showed the highest representation in the clone libraries [36], [37], [50], [51], [52]. On the other hand, this species also showed the highest abundance of clones in *P. persica* roots when integrated pest management treatments (ComFert+IntM and InorgFert+IntM ) were applied (Table 1). Several studies have shown that the presence of *Trichoderma harzianum* significantly increases root colonisation by *Rhizophagus intraradices* in melon crops [53], [54], *R. intraradices* being the only taxon that increased the *T. harzianum* populations [53]. Therefore, a synergistic relationship between *T. harzianum* and *R. intraradices* could have existed in *P. persica* roots under the ComFert+IntM and InorgFert+IntM treatments, increasing their respective abundances.

**Table 1:** Relative abundance of the different AMF sequence types observed in *Prunus persica* roots under the different treatments analysed.

	Treatments			
Phylotypes	ComFert+IntM (n = 60)	InorgFert+IntM(n = 55)	InorgFert+ChemM (n = 49)	ComFert+ChemM (n = 63)
Para 1	13.3	14.6	12.2	11.1
Para 2	3.3	0	4.1	3.2
Glo1	23.3	10.9	8.2	19.1
Glo2	0	0	0	3.2
Glo3	26.7	25.5	16.3	3.2
Glo4	3.3	0	0	0
Glo5	0	0	0	4.8
Glo6	8.3	5.5	6.1	4.8
Glo7	10.0	0	28.6	3.2
Glo8	0	3.6	0	0
Glo9	3.3	0	0	3.2
Glo10	0	9.1	4.1	0
Glo11	0	10.9	0	0
Glo12	0	3.6	16.3	11.1
Glo13	5.0	9.1	0	7.9
Glo14	0	3.6	0	3.2
Glo15	3.3	3.6	4.1	9.5
Glo16	0	0	0	3.2
Aca 1	0	0	0	3.2
Scu 1	0	0	0	3.2
Arch 1	0	0	0	3.2

ComFert: Combination of organic and inorganic fertilization; IntM: Integrated pest management; InorgFert: Inorganic fertilization; ChemM: Chemical pest control.

Interestingly, in contrast to Lee et al. [55] who reported that the AML1/AML2 primer pairs do not amplify sequences belonging to the family *Archaeosporaceae*, we detected the Arch 1 phylotype, which showed 99% homology with sequences related to *Archaeospora trappei*.

There were phylotypes that occurred exclusively in some treatments; for example, Glo 2, Glo 5, Glo 16, Aca 1, Scu 1 and Arch 1 seemed to be specific for trees treated with ComFert+ChemM. The low abundance and specificity found for these phylotypes could be attributable to different colonisation strategies by these different phylogenetic groups. For example, it has been reported that mycelia of *Acaulospora* species have low root and soil colonisation levels [56]

Phylotypes Glo 8 and Glo 11 only appeared in the roots of trees receiving the InorgFert+IntM treatment and Glo 4 occurred exclusively with the ComFert+IntM treatment. The remaining phylotypes did not have a clear distribution, occurring haphazardly in two or three treatments, as in the case of Glo 14, related to sequences belonging to *Funneliformis mosseae.* Although in our study this phylotype was of low abundance (6.81%), this taxon appears to be one of the most-typical and dominant taxa in many agricultural fields [34], [35], [37], [47], [57]. Together with *R. intraradices*, these taxa are sometimes called the “typical AMF of arable lands” [47].

## Conclusions

The real causes and effects of these differences in the AMF community composition observed among treatments are very difficult to establish, bearing in mind that the different crop management regimes studied consist of several influencing parameters. In fact, the ComFert+ChemM - which produced the highest diversity of AMF - included the highest number of parameters. Further investigation of AMF diversity, including analysis of each factor separately and subsequently their interactions, could help to ascertain the cause of the effects reported here.

Different crop management strategies can exert a clear influence on the populations of AMF. The treatment including a combination of organic and inorganic fertilisation together with chemical pest control appears to be the most-suitable with respect to improve of the AMF diversity in this crop under tropical conditions, thus improving the agricultural and environmental sustainability of this agro-ecosystem.

## Supporting Information

File S1 **Supporting tables. Table S1.** AMF sequences obtained in the present study for each phylotype. **Table S2.** Chemical properties of soil in response to different treatments analysed at the time of sampling (n = 4).
(DOC)
