# Supplementary material for: Prunus persica Crop Management Differentially Promotes Arbuscular Mycorrhizal Fungi Diversity in a Tropical Agro-Ecosystem
Source: PLoS One. 2014 Feb 10;9(2):e88454. doi: 10.1371/journal.pone.0088454 (PMC3919774; doi:10.1371/journal.pone.0088454)
Supplement: File S1 — Supporting tables. Table S1. AMF sequences obtained in the present study for each phylotype. Table S2. Chemical properties of soil in response to different treatments analysed at the time of sampling (n = 4). (DOC) [file pone.0088454.s001.doc]

**SUPPORTING INFORMATION S1**

| **Table S1.** AMF sequences obtained in the present study for each phylotype | | | | |
| --- | --- | --- | --- | --- |
|  | **Treatments** | | | |
| **Phylotypes** | **T1** | **T2** | **T3** | **T4** |
| **Para 1** | T1.1-13, T1.1-15, T1.2-8, T1.2-12, T1.2-13, T1.2-22, **T1.3-5**, T1.3-14, | **T2.1-3**, T2.1-7, T2.1-9, T2.1-11,  T2.1-12,  T2.2-1,  T2.2-14,  T2.4-17 | T3.1-32, **T3.2-2**, T3.2-28, T3.3-2, T3.3-22, T3.4-12 | T4.1-2, **T4.1-11**, T4.1-13, T4.2-2, T4.2-6, T4.2-13, T4.3-4 |
| **Para 2** | **T1.3-2**, T1.2-5 |  | **T3.3-15**, **T3.4-6.** | **T4.3-5**, T4.4-14 |
| **Glo1** | T1.1-1, T1.1-2,  T1.1-3, T1.1-5, T1.1-6, T1.1-9,  T1.1-10, T1.1-12, T1.1-14, T1.1-16, T1.2-1, **T1.2-11**, T1.3-8, T1.3-11 | T2.1-15, **T2.3-4,** T2.3-5, T2.3-9, T2.3-10, T2.4-12, | T3.2-3, **T3.2-4,** T3.2-21, T3.2-26 | T4.1-8, T4.1-15, T4.2-1, T4.2-4, T4.2-12, T4.3-6, T4.3-7, T4.3-8, T4.3-11, T4.3-12, **T4.3-13**, T4.3-16 |
| **Glo2** |  |  |  | **T4.3-15**, T4.4-21 |
| **Glo3** | T1.1-8, T1.2-3, T1.2-15, **T1.2-21,** **T1.3-6**, T1.4-1, T1.4-2, T1.4-3, **T1.4-7**, T1.4-9, T1.4-4, T1.4-5, T1.4-8, T1.4-10, T1.4-13, T1.4-14 | **T2.1-10**, T2.1-17, T2.3-16, T2.3-18,  T2.4-2, T2.4-8, T2.4-9,  T2.4-1, T2.4-10, T2.4-11, T2.4-13, T2.4-14, T2.4-15, T2.4-16, | **T3.2-10**, T3.2-20, T3.2-22, T3.2-24, T3.2-27, T3.2-29, T3.2-30, **T3.2-19**, | **T4.1-1**, T4.3-19 |
| **Glo 6** | **T1.1-7**, T1.1-17, T1.2-16, T1.3-4, T1.3-9, | **T2.2-2**, T2.2-12, T2.2-15 | **T3.2-1**, **T3.3-9**, T3.3-17 | T4.2-9, T4.3-9, **T4.1-16**. |
| **Glo 4** | **T1.2-6,** T1.3-7 |  |  |  |
| **Glo 5** |  |  |  | **T4.4-9, T4.4-17,** T4.4-30. |
| **Glo 7** | T1.3-12, T1.3-15, **T1.3-16**, T1.4-6, T1.4-11, T1.4-15 |  | T3.1-1, T3.1-4, T3.1-14, T3.1-23, T3.1-30, T3.4-2, T3.4-3, T3.4-7, T3.4-8, T3.4-9, T3.4-10, **T3.4-11**, T3.4-15, T3.4-16, | **T4.4-5**, T4.4-32 |
| **Glo10** |  | T2.1-14, T2.2-9, T2.3-6, T2.3-11, **T2.4-4** | **T3.1-11**, T3.2-14 |  |
| **Glo 8** |  | **T2.2-8**, T2.1-27 | . | . |
| **Glo 9** | **T1.4-12**, T1.4-32 |  |  | **T4.4-20**, T4.3-3 |
| **Glo 11** |  | **T2.2-3,T2.2-6**, T2.2-7, T2.2-10, T2.2-11, T2.2-13 |  |  |
| **Glo 12** |  | T2.1-4, **T2.1-5**, | T3.3-4, **T3.3-8**, T3.3-11, T3.3-20, T3.3-23, T3.3-30, T3.3-31,T3.3-32 | T4.4-3, T4.4-4, T4.4-13, T4.4-18, T4.4-27, T4.4-29, **T4.4-8** |
| **Glo 13** | **T1.2-4**, T1.2-9, T1.2-10 | **T2.1-2,** **T2.4-5,** T2.4-6, T2.1-1, T2.1-16 |  | T4.1-3, **T4.1-4**, T4.1-5, T4.1-9, T4.1-14 |
| **Glo 14** |  | **T2.3-1,** T2.3-12 |  | T4.4-1, **T4.4-28** |
| **Glo 15** | **T1.3-10**, T1.2-32 | **T2.3-15**, T2.1-31 | **T3.3-7**, T3.1-27 | **T4.2-3**, T4.2-10, T4.2-11, T4.3-2, T4.2-8, T4.2-16 |
| **Glo 16** |  |  |  | **T4.1-7**, T4.3-32 |
| **Aca 1** |  |  |  | **T4.2-15**, T4.3-22 |
| **Scu 1** |  |  |  | **T4.3-1**, T4.1-19 |
| **Arch 1** |  |  |  | **T4.2-7**, T4.2-14 |
|  |  |  |  |  |

| **Table S2.** Chemical properties of soil in response to different treatments analysed at the time of sampling (n=4) | | | | |
| --- | --- | --- | --- | --- |
|  | **Treatments** | | | |
|  | **ComFert+IntM** | **InorgFert+IntM** | **InorgFert+ChemM** | **ComFert+ChemM** |
| pH (H_2_O) | 5.2a | 5.1a | 5.1a | 5.1a |
| N (gKg^-1^) | 3.70a | 3.51a | 3.61a | 3.67a |
| P (gKg^-1^) | 48.2a | 47.4a | 47.8a | 49.1a |
| K (gKg^-1^) | 132a | 128a | 130a | 135a |
| ComFert: Combination of organic and inorganic fertilization; IntM: Integrated pest management; InorgFert: Inorganic fertilization; ChemM: Chemical pest control. Values in row followed by the same letter do not differ significantly (*P* < 0.05) as determined by the LSD test. | | | | |
|  | | | | |
